# Appendicitis to multivisceral transplantation: a career experience with appendiceal malignancy

**DOI:** 10.1308/rcsann.2023.0013

**Published:** 2023-06-27

**Authors:** BJ Moran

**Affiliations:** Hampshire Hospitals NHS Foundation Trust, UK

**Keywords:** Multivisceral, Transplantation, Career, Experience, Appendiceal

## Abstract

John Hunter is regarded as the father of scientific surgery. His principles involved reasoning, observation and experimentation. His most powerful saying was: “Why not try the experiment?” This manuscript charts a career in abdominal surgery ranging from the treatment of appendicitis to the development of the largest appendiceal tumour centre in the world. The journey has led to the first report of a successful multivisceral and abdominal wall transplant for patients with recurrent non-resectable pseudomyxoma peritonei. We all stand on the shoulders of giants and surgery progresses by learning from the past while being prepared to experiment into the future.

## Introduction

John Hunter (1728–1793) is generally regarded as the father of scientific surgery and his lifetime achievements are an inspiration to all who pursue a career in surgery. An excellent biography by Wendy Moore summarises John Hunter’s unconventional training with his progression from self-taught anatomist to the most skilled surgeon of his generation and outlines many of his numerous scientific experiments along the way.^1^ *The Knife Man* also provides an insight into the origins of one of the most famous medical museums in the world, the Hunterian Museum at the Royal College of Surgeons of England (RCS England), which has been extensively refurbished and reopened in spring 2023.

Born in 1728 on a Scottish farm, John Hunter was the younger brother of William Hunter, himself an anatomist, surgeon and eminent gynaecologist. Both brothers are remembered for posterity by a Hunterian museum: John at RCS England and William at the University of Glasgow. John Hunter died in 1793 from a myocardial infarction said to be brought on by a rage while attending a medical management board meeting, perhaps a lesson for us all in the futility of rage towards medical management. In my opinion, it is better to engage and work with management than to confront and antagonise.

John had no formal education as such, and his first exposure to anatomy and surgery was as an apprentice to his established brother, William, who had set up an anatomy dissection and surgical training school in London. Quickly, John learnt the skills of anatomical dissection, applying those skills to a lifetime interest in dissecting and preserving animals (from dogs and cats to lizards, crocodiles and elephants), all types of plants, and numerous human anatomical specimens, many of which are preserved to this day.

John Hunter is renowned for challenging the status quo and “trying the experiment”. He pursued his lifetime interest in dissecting human and animal bodies to advance the knowledge of anatomy, the process of life and the causes of death. He believed that surgery should be based on the scientific principles of reasoning, observation and experimentation.

Some of his methods would be considered dubious by today’s standards, as indeed some of our own current efforts may well be when examined 100 years from now. For instance, Hunter performed some placebo-controlled experiments without informing the subjects that he was doing so. One famous example was when he compared “bread pills” with mercury pills for the treatment of sexually transmitted diseases. Hunter questioned the dogma of the day and felt that sexually transmitted diseases were generally self-limiting, and that all the pills and potions and bizarre remedies were of no benefit (and might even be harmful), a recurring theme in many medical and surgical interventions throughout history. Needless to say, many of his fellow practitioners were appalled that he should doubt their “miracle cures” and he was loathed for his “unconventional” ideas.

Like all of us who practise medicine and surgery, John Hunter did not always get it right; he was convinced that gonorrhoea and syphilis were the same disease. He described the symptoms and signs following deliberate infection of a man (almost certainly himself) by puncturing the penile skin with a sharp instrument that he had deliberately contaminated from a patient with venereal disease. He carefully documented the symptoms and signs, and concluded that gonorrhoea and syphilis were one disease, publishing extensively on his results. However, he had unwittingly infected the subject (probably himself) with both organisms. His conclusion put back the knowledge around sexually transmitted diseases by several decades.^1^

Hunter is also reputed to have performed an elegant (albeit unethical) randomised trial by substitution of water from the Thames for his wife’s expensive bottled water. He filled the empty bottles with the polluted river water and noted that his wife could not detect the difference.

John Hunter’s attention to surgical detail and thought processes around surgical intervention were controversial although generally well founded on reasoning, observation and experimentation. Concerning these three facets, he was meticulous in recording results of his observations and experiments. He established that tying off the common femoral artery in what we now call Hunter’s canal on the medial aspect of the thigh, well above a popliteal aneurysm, resulted in far better results than the alternatives at that time. The standard (direct) approach to a popliteal aneurysm almost inevitably led to fatal uncontrollable haemorrhage. For this reason, the other alternative of a leg amputation was favoured by many, with overwhelming risk of death from postoperative sepsis if the unfortunate patient did not die on the operating table having an amputation without anaesthesia.

During Hunter’s time as a war surgeon, he recognised that battlefield surgery was almost always fatal and proposed a non-operative approach for soldiers with embedded fragments of metal where “surgical probing” was (at that time) followed unquestioningly. He noted that a small group of enemy soldiers who had hid for several days with shrapnel injuries were alive and well without surgical probing. He also noted the consequences of probing: probing being performed without anaesthesia or antibiotics; the contaminated, unwashed hands of the surgeons spreading lethal infections from patient to patient; and the agonising deaths.

John Hunter’s most prized specimen was the skeleton of the “Irish Giant”, a man called Charles Byrne, who probably had acromegaly from a pituitary tumour. He is estimated to have been approximately 7’ 7” tall, and died in his early twenties from a combination of disease and overindulgence of alcohol.

## Standing on the shoulders of giants: the giants in my surgical career

Isaac Newton (1642–1727) is reputed to have first used the term “standing on the shoulders of giants”, a saying engraved on the rim of the British £2 coin. John Hunter and Charles Byrne were both giants in their different ways, and for all of us in surgery, there are giants who influenced and (helped forge) our training and careers. The three surgical giants in my career have been Pat Malone,^2^ Bill Heald^3–5^ and Paul Sugarbaker,^6,7^ who have shaped my career over the past three decades.

My life with the appendix began in 1980 in Limerick Regional Hospital (now University Hospital Limerick) in Ireland. I commenced as a surgical intern (F1 equivalent) on 1 July 1980. Pat Malone was my immediate boss as a senior house officer and I would assist Pat in performing the numerous appendicectomies in an era where there was no computed tomography (CT), magnetic resonance imaging (MRI) or ultrasonography available. Indeed, my recollection was that anyone passing nearby with abdominal pain usually finished up having an appendicectomy. In addition, a patient with chronic abdominal pain who presented repeatedly to a surgeon would have been listed for the then well established “diagnostic laparotomy”.

In the current era of cross-sectional imaging and laparoscopy, this would now be regarded as barbaric but in the 1980s, it was practised widely. A right paramedian incision was performed, with full inspection and palpation of the abdominal cavity. If all was well, an appendicectomy and commonly also a cholecystectomy for good measure were performed. Removal of these organs (almost always normal) reassured most patients and the surgeon for some time although the original pain was often replaced by the lifelong curse of adhesion related pain and occasionally, life threatening bowel obstruction.

Having assisted Pat at a few appendicectomies, the time came for me to be the surgeon and Pat the assistant. Through a small Lanz (gridiron) incision in the right lower quadrant, the peritoneum is eventually reached, grasped with two forceps and opened using a scalpel or scissors. The index finger of the right hand was introduced to “hook” the caecum and the appendix. Finding and removing a perforated appendix, and observing the rapid recovery over the next few days meant I was “hooked” for life on abdominal surgery, and ultimately, on the journey from appendicitis to appendiceal tumours, culminating in multivisceral transplantation.

Pat Malone progressed to global fame from his base in Southampton as an eminent paediatric surgeon. Pat developed a new operation using the appendix, called the Malone antegrade continence enema (MACE) procedure, for the treatment of faecal incontinence or intractable constipation in children.^2^ The MACE was adapted from the Mitrofanoff procedure.^8^ It was subsequently adopted internationally in both children and adults, with modifications for patients without (or with an inadequate) appendix, and continues to be used in selected cases.

The original MACE involved reversing the appendix and reimplanting the tip in the caecum with the appendix base sutured to the skin so that a catheter could be inserted to flush out the colon. This concept developed by Pat has helped improve the quality of life for many. It is an inspiration for all of us to follow Hunter’s concept of reasoning, observation and experimentation when designing and developing a novel technique.

My own career took me from Ireland to Ghana, West Africa, as a medical volunteer with my wife Karina, whom I had met when we were both senior house officers in Limerick (Karina in general medicine and I in surgery). In Ghana, management of obstructed labour was a major part of our emergency workload. My first ever publication in the literature was a letter in *The Lancet*, where I challenged the dogma of the time that antibiotics were not needed for Caesarean section and proposed that all women having emergency Caesarean section should receive prophylactic antibiotics to reduce the septic postoperative complications.^9^

It was notable that with an annual caseload of over 80,000 hospital attendances and numerous emergency procedures, we did not encounter a single case of appendicitis in Ghana over the two-year period we were there in the mid-1980s.

## The role of the appendix, appendicitis and appendicectomy

For most surgeons of my generation, an appendicectomy was the first abdominal operation that we performed. When asked what the appendix does, I usually reply: “Not a lot!” – unless it gives trouble by inflammation or (rarely) malignant transformation. I usually add that the main role of the appendix is to provide a training operation for surgeons, in my era by open surgery and currently, by laparoscopic appendicectomy. However, as time has passed, we now appreciate that the appendix has an immune function and may also be a sanctuary site for the human microbiome, in addition to its role as a conduit to the colon and bladder, as described by Malone^2^ and Mitrofanoff^8^ respectively. Research continues on the effect of appendicectomy preventing (or alleviating) ulcerative colitis as an immune regulator or altering the microbiome, or perhaps a combination of both.

Appendicitis is one of the most common surgical emergencies but there are still questions relating to optimal treatment. Appendicectomy remains the gold standard but as with the management of sigmoid diverticulitis, treatment of diverticulitis or appendicitis by antibiotics alone (or indeed without antibiotics) has been proposed and evaluation is ongoing.

Traditionally, appendicectomy was the only accepted treatment for appendicitis, with conservative treatment by intravenous antibiotics for “an appendix mass” where emergency appendicectomy was considered unsafe. Interval appendicectomy was recommended at 3–6 months after resolution of the appendix mass. As outlined in my early surgical life, prior to cross-sectional imaging, appendicectomy was undertaken more liberally, with acceptance that the appendix would be histologically normal in a substantial proportion of cases. The widespread availability of abdominal imaging (particularly CT) has facilitated more accurate diagnosis of appendicitis and has been the baseline for a number of recent good quality randomised trials.^10^ In this context, CT has allowed categorisation of appendicitis into “uncomplicated” and “complicated”, the latter including perforation, abscess formation and appendix mass.

There is almost universal agreement that a patient with a perforated appendix with localised or generalised peritonitis should undergo appendicectomy (by a laparoscopic or open approach). Nevertheless, controversy persists with regard to the management of uncomplicated appendicitis although a 2022 meta-analysis of operative versus non-operative management of acute appendicitis is informative, with 18% of the non-operative group developing subsequent appendicitis.^10^ Indeed, a five-year follow-up of patients randomised to non-operative management for appendicitis reported that almost 40% of those treated by antibiotics developed further appendicitis.^11^

There is also ongoing debate as to whether patients with complex appendicitis diagnosed by CT require interval appendicectomy if the symptoms resolve on conservative treatment. A Finnish group developed and initiated an elegant randomised trial on the need for interval appendicectomy for patients with a CT diagnosis of an appendix mass or abscess treated by non-operative treatment.^12^ They aimed to recruit 100 patients randomised to interval appendicectomy or to follow-up using abdominal MRI. At interim analysis, after 60 patients had been randomised, the trial was closed owing to the risk of appendiceal malignancy, which was 20% (12/60) overall and 35% (12/34) in patients over the age of 40 years. Table 1 gives a brief overview of the current state of appendicitis and its management.

**Table 1:** Summary of the current state of appendicitis and its management

– Mild appendicitis is common, underdiagnosed and can settle spontaneously. – Non-complicated appendicitis diagnosed on computed tomography can be treated with antibiotics but severe recurrent acute appendicitis may occur in 18–40% of patients by five years.^10,11^ – An appendix mass/abscess diagnosed on computed tomography and treated non-operatively has a risk of underlying appendiceal malignancy of 7–20% and interval appendicectomy should be considered.^12^

## Basingstoke: total mesorectal excision and the first pseudomyxoma cytoreductive operation

My general surgical training in England commenced in 1986 as surgical registrar for a year at Queen Mary’s Hospital in London, followed by two years of research in human nutrition in Southampton. I completed a master’s degree on the function of the human colon and summarised my findings in a *British Journal of Surgery* review.^13^

I was fortunate to obtain a surgical rotation to Basingstoke, arriving there in 1989 on the ascendency of total mesorectal excision, described and popularised by the legendary Bill Heald.^3–5^ Bill’s pioneering work on rectal cancer has laid the foundations for one of the biggest advances in surgical oncology of my lifetime, and the concepts of total mesorectal excision and the “holy plane” are the bedrock of optimal management for rectal cancer, whether surgery is open, laparoscopic, robotic or transanal.

In 1994, I returned to Basingstoke as what was then termed a senior registrar, prior to eligibility to apply for a post as a consultant surgeon. During my year as a senior registrar, Bill Heald was asked for an opinion on a young man from Scotland called Brian, who had an “open and close” laparotomy for pseudomyxoma peritonei (PMP), something that none of us felt we had ever seen in our many years in surgery. In retrospect, we had almost certainly encountered several cases, usually labelled as diffuse carcinoma of unknown origin or pancreatic carcinomatosis and commonly discovered at open and close laparotomy in an era prior to imaging.

Bill remembered that he had heard a presentation on PMP from another surgical giant, Paul Sugarbaker, from Washington. On invitation, Paul agreed to join us for this case and in March 1994, we completed the 14-hour operation in Basingstoke with complete tumour removal combined with intraperitoneal chemotherapy in the form of mitomycin C at 10mg/m^2^.

Brian survived ten years but died ultimately from inoperable abdominal recurrence. One of the most remarkable aspects of this case was that we managed to extract £40,000 from Scotland to cover the cost of the procedure.

## From case 1 to thousands: the development of a global appendiceal tumour centre

The second case of PMP was referred a few months before I was appointed as a consultant general surgeon in Basingstoke in 1996. Bill had deferred seeing her until I arrived in October. Teresa had had two previous laparotomies and multiple rounds of systemic chemotherapy, and was considered inoperable with no further treatment options. She had tried to get National Health Service (NHS) funding for treatment in Washington by Paul Sugarbaker but the cost of $100,000 was prohibitive. Sugarbaker suggested she try Basingstoke.

Teresa was booked to see me in my first outpatient clinic. She asked me how many of these procedures we had done and what made me think we could do surgery for PMP. I said that Bill and I had assisted at one case, that we knew the principles of the procedure and that we were prepared to operate if she was willing to risk it. In November 1995, we completed her operation in 14 hours with complete tumour removal. She is alive and well 27 years later, with no further treatment and no recurrence. The next case was straightforward surgically although complicated by a postoperative fistula with four weeks in hospital.

The fourth case was a gamechanger owing to the unforgettable disastrous complications. The patient had uncontrollable bleeding from the liver after liver capsulectomy with six reoperations for bleeding (including 4 in the middle of the night). The total cumulative blood transfusion was 164 units of blood. Nevertheless, he survived and was discharged home after 80 days in hospital including 60 days in the intensive care unit. Our hospital management team had agreed a treatment tariff of £25,000 with the referring hospital in Birmingham and suddenly, the costs of treatment were estimated to be at least £150,000. Birmingham stood firm and it was apparent that PMP surgery had moved from “winner” to “loser”, from a good income generator to certain bankruptcy, if we were to continue with these cases without adequate funding.

This fourth case stimulated an application for funding for PMP assessment and treatment in Basingstoke to the National Specialist Commissioning Advisory Group, which was an NHS subsidiary with ability to centrally fund rare disease treatment. The bid to treat PMP in 1999 was initially refused but was successful in 2000 when I specified treatment of PMP of appendiceal origin and that the incidence was one in a million population per year.^14^ What is now the Basingstoke Peritoneal Malignancy Institute was commissioned by the English NHS for treatment of PMP in 2000 and a second centre was opened in Manchester in 2002.

The outcomes of the first 100 cases were carefully documented and details published^15^ but perhaps more importantly, the experience of the learning curve was described.^16^ The first 100 cases were divided into 33, 33 and 34 cases, and the significant reduction in mortality (from 18% to 3%) and major morbidity were noted to be predominantly around decision making (particularly case selection), in combination with technical errors in the earlier experience.

Subsequently, an increasing referral base allowed reporting of outcomes on 1,000 patients with appendiceal tumours treated with a combination strategy of cytoreductive surgery (CRS) and hyperthermic intraperitoneal chemotherapy (HIPEC).^17^ To date, we have treated more than 2,000 patients with appendiceal tumours and have translated the strategy of CRS and HIPEC to more common resectable peritoneal malignancies such as colorectal peritoneal metastases.^18^

**Figure 1:**
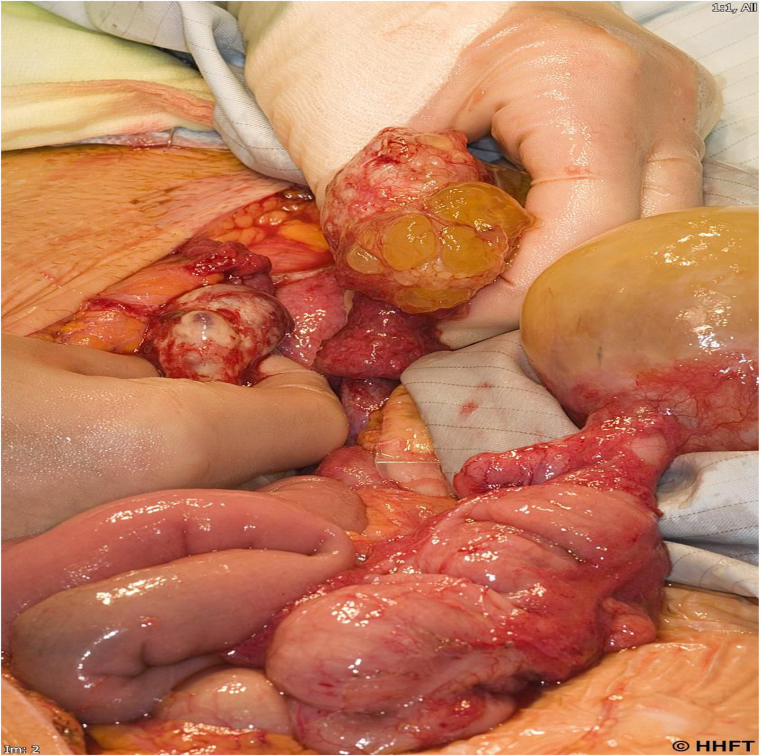
Mucinous tumour of the appendix with ovarian metastases

Tumours of the appendix are rare but increasing (Figure 1). An estimate from 2021 suggests a much higher incidence rate (3.2 people per million per year) than my original estimate of one per million per year.^19^ We have recently reported that appendiceal tumours present in many diverse ways and often coincidentally at cross-sectional imaging, or at laparoscopy or laparotomy.^20^

**Figure 2:**
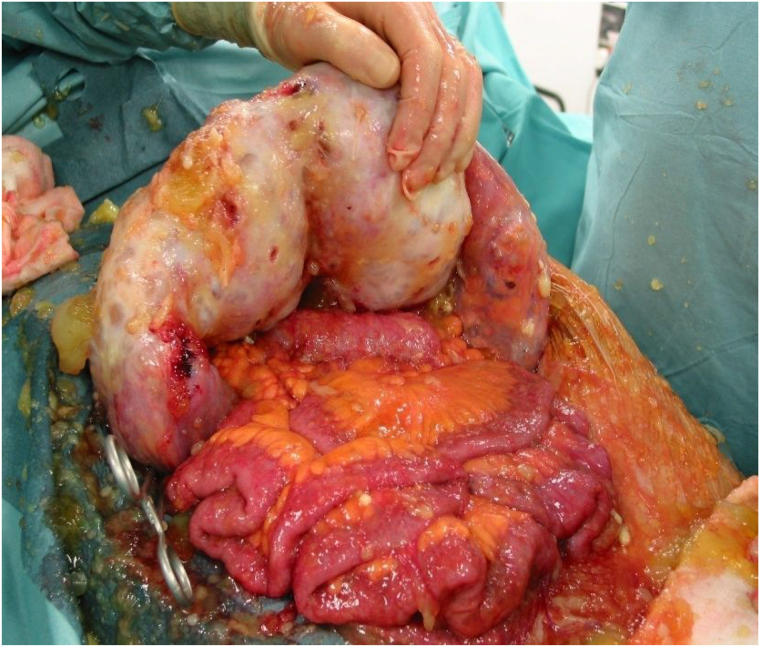
Omental cake

The paradox of PMP is that the most extensive procedures may be needed for what are often the least pathologically aggressive neoplasms. In this context, in our experience, approximately 10% of PMP patients undergoing CRS and HIPEC require a partial gastrectomy, and (often simultaneously) a total colectomy and end ileostomy. We also introduced the strategy and terminology of “major tumour debulking”, generally involving a greater omentectomy and subtotal colectomy, when complete tumour removal is not possible, usually because of extensive small bowel involvement (Figure 2).^21^

## Current major dilemmas in PMP management

The management of patients with PMP who cannot have complete cytoreduction and of those who develop recurrence after complete cytoreduction are the biggest challenges. It is well documented from a multicentre experience with over 2,000 cases^22^ and from our own experience with 1,000 cases^17^ that one of the main determinants of outcome is complete tumour removal by CRS. We have demonstrated that our complete cytoreduction rate increased over time with increasing experience.^23^ Nevertheless, there remain many cases where extensive involvement of the small bowel precludes complete tumour removal.

Additionally, in all reports,^22,23^ despite complete cytoreduction, over 30% of PMP cases recur. In 2021, we published the largest single centre experience with recurrence in over 400 patients out of 1,100 who had complete tumour removal at the primary operation.^24^ We operated on some, experimented with systemic chemotherapy and immunotherapy for many but have few solutions for most.

One of the unique features of PMP is that many cases are histologically low grade, with disease recurrence and progression occurring almost exclusively within the abdomen by local progression. Ultimately, intestinal failure from bowel compression and invasion of the abdominal wall and other organs is inevitable. Intestinal failure is often accompanied by abdominal wall failure, whereby tumour growth in the abdominal wall, and fistulation of mucus and bowel contents occurs, with no systemic metastatic disease.

## Multivisceral and abdominal wall transplantation for non-resectable PMP

**Figure 3:**
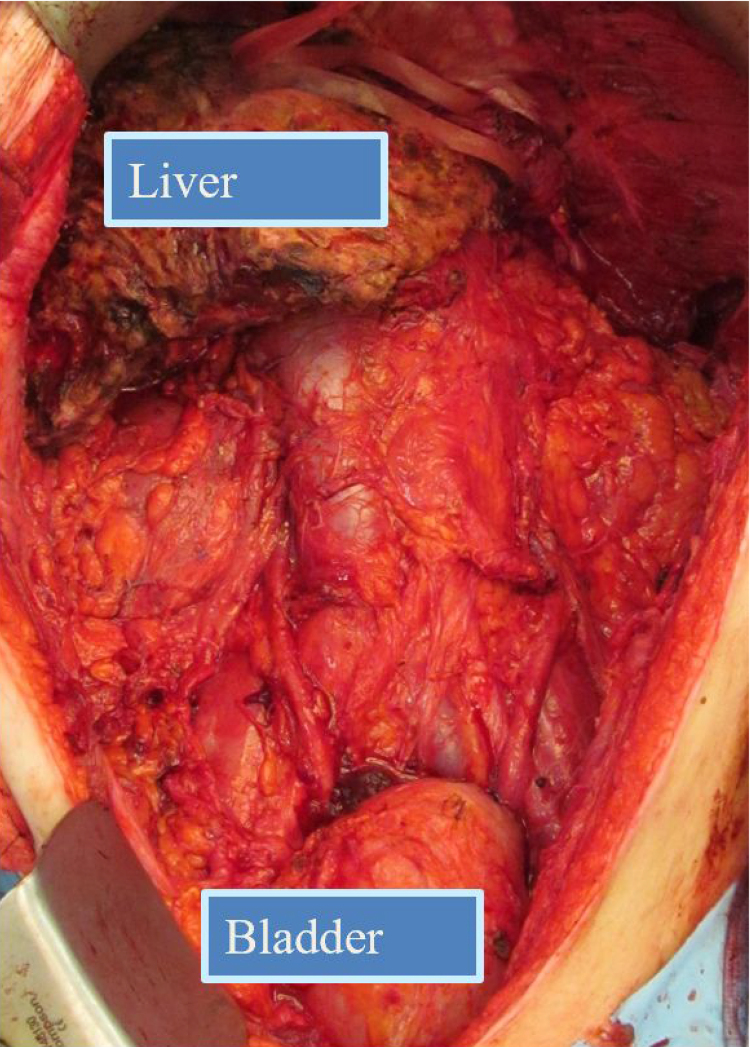
Empty abdomen after tumour removal prior to multivisceral transplant

In 2013, Steve Prescott, a patient of ours who had major tumour debulking in 2006, and whose disease had progressed with abdominal wall and intestinal failure, challenged the Basingstoke peritoneal malignancy team and the Oxford transplant team to remove his tumour and abdominal wall (as in the intraoperative photo of Adam, a subsequent patient, Figure 3), combined with cadaveric multivisceral transplantation and an abdominal wall transplant. Steve had been an international rugby league player. His memory lives on in his autobiography, completed by his wife Lindsey,^25^ and in the annual Steve Prescott Man of Steel award to the top rugby league player in England.

We wondered about transplantation but did we dare? Even if achievable, would the anti-rejection drugs result in massive tumour regrowth? No one knew but as John Hunter would say: “Why not try the experiment?” The word “transplant” may well have been first used by John Hunter. Hunter had taken healthy teeth from young paid “donors”, “planting” them in the gums of older people who had lost their teeth owing to tooth decay, a consequence of a surge in the intake of the newly available large quantities of sugar at the time. Hunter and others reported that tooth transplantation was feasible and worked for a few years although inadvertent transmission of venereal and other diseases was a hazard. Nevertheless, tooth transplantation became fashionable for many years thereafter.

In the summer of 2013, Steve Prescott underwent a multivisceral and abdominal wall transplant but died within 90 days from overwhelming sepsis and organ rejection. In retrospect, like many pioneering first organ transplants, the recipient’s general condition (and the extent of the disease) is usually associated with failure. However, Steve’s bravery and persistence had established proof of principle, and we have just reported on our combined experience with Oxford on abdominal wall and multivisceral transplantation in 15 patients with recurrent non-resectable PMP.^26^ The actuarial one-year and five-year survival rates are 79% and 55% respectively. Being a part of this advance remains one of the highlights of my surgical career.

## Conclusions

This simple vestigial organ, the vermiform appendix to give it its true title, has taken me on an exciting surgical journey commencing with my first abdominal operation, and culminating in the world’s first successful multivisceral and abdominal wall transplant for recurrent non-resectable PMP. Along the way, the journey has been punctured by joy and sorrow, triumph and disaster, and has been driven and facilitated by innumerable health professionals as well as brave adventurous patients. It has also been a privilege to mentor and support international CRS and HIPEC units in Ireland^27^ and Australia,^28^ and to lead and develop global guidelines.^29^ I hope that John Hunter would feel that we have tried to follow his principles of reasoning, observation and experimentation, and that surgery will continue to evolve by these routes, and by documentation of techniques and outcomes.

## Acknowledgement

This paper is based on a Hunterian lecture delivered at the Annual Meeting of the Association of Coloproctology of Great Britain and Ireland, held in Edinburgh in July 2022.
